# CompareSVM: supervised, Support Vector Machine (SVM) inference of gene regularity networks

**DOI:** 10.1186/s12859-014-0395-x

**Published:** 2014-11-30

**Authors:** Zeeshan Gillani, Muhammad Sajid Hamid Akash, MD Matiur Rahaman, Ming Chen

**Affiliations:** Department of Bioinformatics, College of Life Sciences, Zhejiang University, Hangzhou, 310058 China; Institute of Pharmacology, Toxicology and Biochemical Pharmaceutics, College of Pharmaceutical Sciences, Zhejiang University, Hangzhou, China; Faculty of Pharmaceutical Sciences, Government College University Faisalabad, Faisalabad, Pakistan

**Keywords:** Support vector machine, Machine running, Gene regulatory networks, CompareSVM, Supervised learning, Unsupervised learning, CLR (context likelihood to relatedness)

## Abstract

**Background:**

Predication of gene regularity network (GRN) from expression data is a challenging task. There are many methods that have been developed to address this challenge ranging from supervised to unsupervised methods. Most promising methods are based on support vector machine (SVM). There is a need for comprehensive analysis on prediction accuracy of supervised method SVM using different kernels on different biological experimental conditions and network size.

**Results:**

We developed a tool (CompareSVM) based on SVM to compare different kernel methods for inference of GRN. Using CompareSVM, we investigated and evaluated different SVM kernel methods on simulated datasets of microarray of different sizes in detail. The results obtained from CompareSVM showed that accuracy of inference method depends upon the nature of experimental condition and size of the network.

**Conclusions:**

For network with nodes (<200) and average (over all sizes of networks), SVM Gaussian kernel outperform on knockout, knockdown, and multifactorial datasets compared to all the other inference methods. For network with large number of nodes (~500), choice of inference method depend upon nature of experimental condition. CompareSVM is available at http://bis.zju.edu.cn/CompareSVM/.

**Electronic supplementary material:**

The online version of this article (doi:10.1186/s12859-014-0395-x) contains supplementary material, which is available to authorized users.

## Background

The structure and topology of gene regularity network (GRN) is essential to understand the mechanism that how gene transcription factor (TF) regulates genes expression and consequent cellular behaviors such as development, differentiation and response to stimuli. The deregulation of these networks results in change the genes expression and leads to implication in ontogenesis and tumor progression [1]. Technologies like high through output sequencing and microarray offer a great deal of information about individual genes, but the reconstruction of GRN based on genome wide data still remains a big challenge. The prior biological knowledge along with genomics and post genomics data has given rise to supervised techniques to solve this challenge.

Many computational methods have been developed to infer GRN, mostly using unsupervised approaches. Recently, there has been surged in supervised approaches due to identification of large number of transcription factors and their targets which enabled to have sufficient data to train supervised models. The most recent and largest study published by Maetschke SR [2] has clearly shown that the supervised approaches outperform unsupervised and semi-supervised approaches for inference of GRN. They compared the prediction accuracy of 17 unsupervised methods with support vector machine (SVM) and reported it outperform unsupervised methods in different experimental conditions except in knockout experiment by Z-score method. Similar study to evaluate supervised inference of GRN was done by Mordelet and Vert [3]. They compared supervised techniques to context likelihood to relatedness (CLR), algorithms for the reconstruction of accurate cellar networks (ARACNE), relevance networks (RN) and a Bayesian networks (BN) on an *E. coli* benchmark data set by Faith et al. [4].

Any algorithm for supervised learning can be used in principal for interference of GRN. We have used state of art SVM algorithm for inference of GRN for different biological conditions and network size. Ben-Hur and Noble [5] provided a very simple method where a local model is used to estimate the prediction of interacting partners of each protein in the network. This in turn is used for all local models and then combine together to predict edges throughout the network. We used this concept to estimate local model for each TF based on their expression profiles and the genes regulated by TF from other genes. All models were than combined to rank candidate regulatory relationship between TF's and all genes in the genome [3].

In this article, we investigated and compared prediction accuracy of SVM using 4 widely used kernel functions on wide range of networks and experimental data types (multifactorial, knockout, knockdown and all) with comparison to unsupervised methods. The aim of our study was to identify a suitable algorithm for inference of GRN with respect to each experimental condition and network size. We have also developed a simple tool and given the name “CompareSVM” (Supervised inference of Regularity Networks and comparison) for inference and comparison of GRNs (see Additional file 1 to install software and Additional file 2 for instructions how to install and run the software). Although many supervised algorithms have been developed, but we used SVM as it has been successfully applied to inference of GRN. CompareSVM unlike unsupervised techniques requires a list of known regulation relationship between TF and target genes in addition to gene expression data. This is standard method in the field of machine learning for supervised techniques, the known/prior knowledge is used to train a classifier to be able to predict the unseen data. This limitation is of not much concern due to huge surge in the regulation databases for many species and specially availability of well documented model species.

GeneNetWeaver is an open source tool for in *silico* benchmark generation and profiling of network inference. It uses *in vivo* microarray compendia along with synthetic data to access the performance of network inference methods. It enables us to simulate and generate dataset for gene knockdown, knockout and multifactorial microarray expression profiles for *E. coli*. Knockdown experiment refers to the technique in which expression of one or more genes is reduced. Knockout experiment is a genetic technique in which one of the gene expression is completely made inactive and multifactorial experiment refers to the technique where small number of genes expression values are perturbed by a small random number.

We have tested CompareSVM on *E. coli* data extracted by GeneNetWeaver of different nodes (number of genes) ranging from 10 to 500 (see Additional files 3 and 4). Our aim was to identify which inference method worked better for a given experimental condition profiles. We also investigated the accuracy of this method as the size of the network is increased for a certain biological condition profiles. We have identified that for small networks of all biological condition, SVM (Linear, Gaussian, Polynomial) outperform unsupervised inference methods, whereas, for large network SVM ( Linear, Gaussian, Polynomial) also perform better with the expectation of multifactorial experimental condition.

## Methods

### Supervised methods

#### SVM

We used SVM, as it has been successfully applied to infer GRN [3]. We have used SVM library LIBSVM developed by Chih-Chung [6] for implementation of CompareSVM. The essence of SVM is a kernel function *K*(*xi*, *x*) between any two genes x and *xi*, that is the measure of similarity between two genes. In case of GRN, it is measured in terms of expression profiles. Given a set of *m* genes *x*1 … *xm*, SVM estimates a scoring function for any new gene *x* of the form using following equation;

1 $$ f(x)={\displaystyle {\sum}_{i=1}^m\alpha iK\left( xi,\kern0.5em x\right)+\kern0.5em C} $$

**Figure 1:**
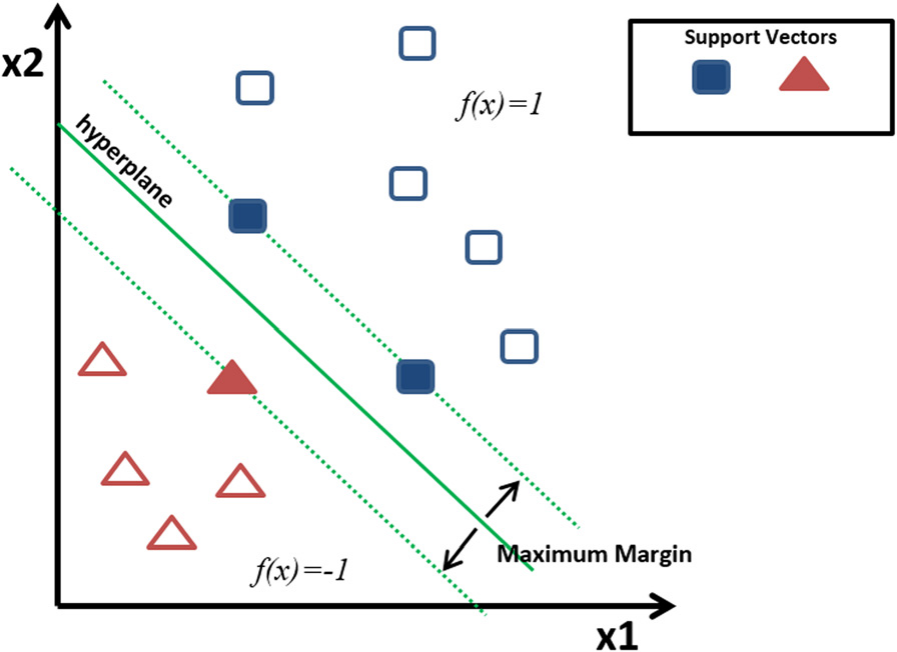
**Two dimensional representation of support vector machine, using maximum margin with support vectors to classify data.** Local model is generated for each transcriptional factor to classify list of genes.

Where, “α*i*” are the weights in the expression to be optimized by the SVM by maximizing the large positive scores for genes in a positive class (+1) and large negative scores for genes in negative class (−1) in the training set. “*C*” is so called complexity parameter that needs to be optimized for predication performance, it also controls possible over fitting of the training set. Once the optimal values of alpha and C are found, genes in the test set can be classified by fitness function *f*(*x*) either to +1 or −1 class as shown in Figure 1.

The kernel *K*(*xi*, *x*) is used by SVM to build scoring function. In our experiments, we want to infer regulation of gene from gene expression profiles of three different biological conditions. Therefore, we represented each collection of gene expression data as a vector. We planned to evaluate the following four kernel functions on four different biological conditions and networks of different size.

- ***Linear kernel***

Linear kernel is simplest kernel function of SVM. It is given by the inner product < *x*, *y* > with addition to *C* constant. Linear kernel is also term as no-linear kernel. Scoring function of the linear is as follow:

2 $$ k\left(x,\kern0.5em y\right)=\left({x}^Ty+c\right) $$

b) ***Polynomial kernel***

Polynomial kernel is nonlinear kernel and has been studied for problem where all the training set is normalized. This makes it ideal for microarray as data is normalized by different normalization techniques before generating expression matrix. Polynomial kernel has two additional parameters, *d* denote the degree of freedom (also known as order of polynomial) and slope of alpha. Scoring function of polynomial kernel is as follow:

3 $$ k\left(x,y\right)={\left(\alpha {x}^Ty+c\right)}^d $$

c) ***Gaussian Kernel***

Gaussian kernel is a radial basis kernel function. It has additional parameter sigma. If overestimated, it will behave almost linear and will lose it non-linearly feature. If underestimated, the kernel will lack regularization and will be highly sensitive to the noise in training set. Scoring function of the Gaussian kernel is as follow:

4 $$ k\left(x,\kern0.5em y\right)= \exp \left(-\gamma {\left\Vert x-y\right\Vert}^2\right) $$

d) ***Sigmoid Kernel***

Sigmoid kernel is also known as hyperbolic tangent (sigmoid) kernel and multilayer perception (MLP) kernel. The origin of this kernel is from theory of neural networks and has been found to be performed well in practice as well. There are two adjustable parameters of sigmoid kernel, slope of alpha and the intercept parameter *C*. Scoring function of sigmoid kernel is as follow:

5 $$ k\left(x,\kern0.5em y\right)= tanh\left(\alpha {x}^Ty+c\right) $$

### Unsupervised methods

The CLR algorithm is an extension of relevance network [7], which predicts regulations between TF and genes by detecting mutual information. CLR was used by Faith [8] for gene network construction from compendium of gene expression data of *E. coli*. CLR uses an adaptive background correction to the estimation of mutual information. Than for each gene, a score is computed for statistical likelihood of mutual information. Then, for each pair of TF-target gene, the mutual information score is compared to the context likelihood of both the TF and the target gene, and turned into a z-score. TF-gene interaction is then ranked by decreasing order [3]. We have used Pearson and Spearman estimation for calculating prediction accuracies for CLR using minet [9] package of R (see Additional file 5).

### CompareSVM

**Figure 2:**
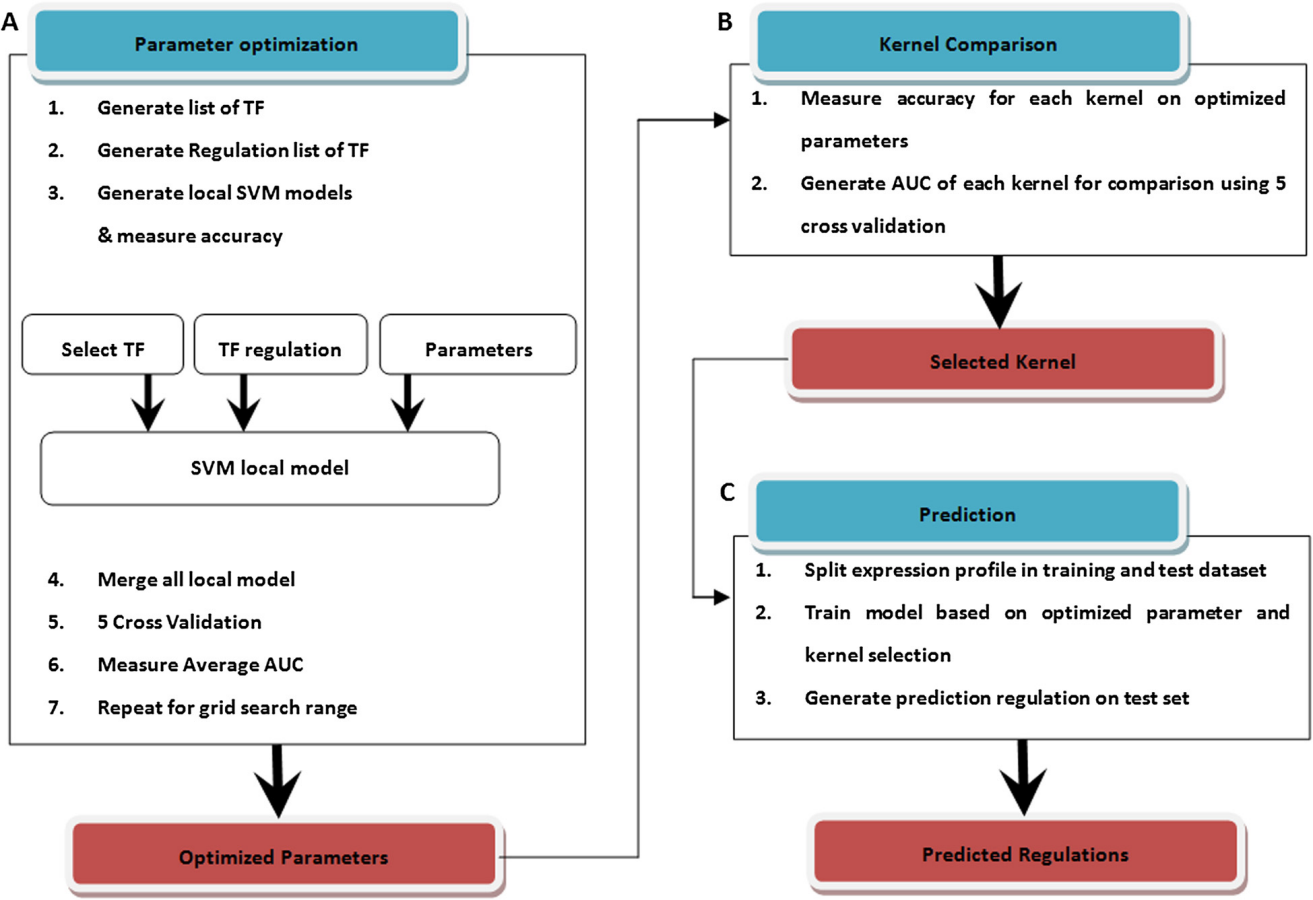
**Typical workflow for inference of gene regularity network using CompareSVM.** 3 section: optimization **(A)**, comparison **(B)** and predication **(C)**.

**Figure 3:**
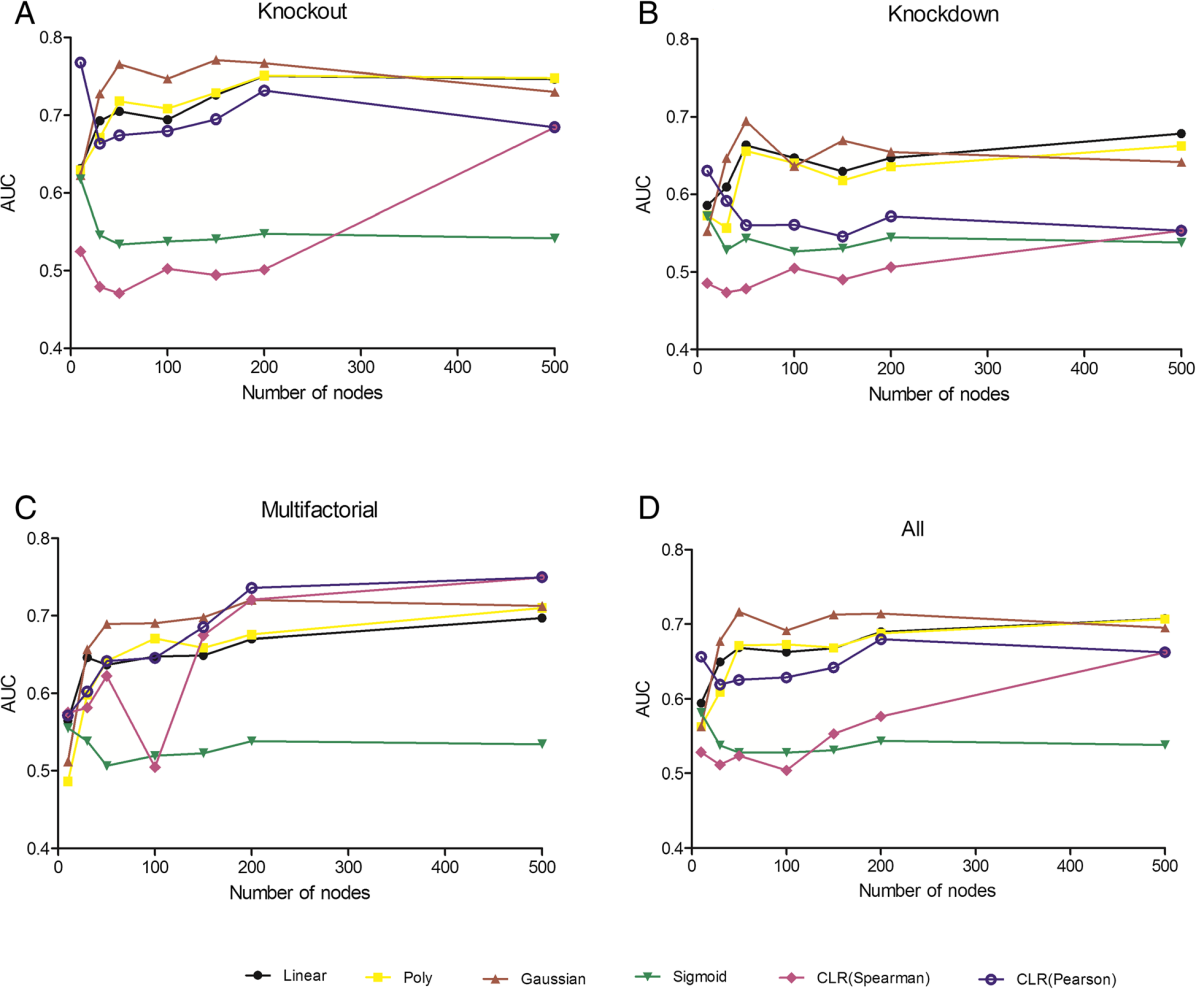
**Prediction accuracy (AUC) of unsupervised and supervised methods on knockout (A), knockdown (B), multifactorial (C) and average (all) data (D) generated by GeneNetWeaver extracted from** ***E. coli.*** For each network of size: 10, 30, 50, 100, 150, 200 and 500. 10 networks were generated for each size and experimental condition.

CompareSVM is implemented in MATLAB, a typical workflow contain 3 sections including optimization, comparison and prediction as shown in Figure 2. Firstly, these parameters are optimized for a given kernel using Grid search in CompareSVM optimization as shown in Figure 2a. For each kernel, two parameters are optimized with the exception of linear kernel, which only require 1 parameter for model optimization. This in turn can be repeated for all kernels. Once parameters are optimized, CompareSVM comparison can generate AUC for each kernel as shown in Figure 2b. The kernel with higher accuracy and its optimized parameters can be used in CompareSVM prediction to identify new targets of TF (Figure 3). If the optimized parameters are already known, the CompareSVM prediction can be used directly.

CompareSVM is a SVM tool to infer new regulation between list of TF and all genes in the organism. It requires two types of inputs. Firstly the list of genes and their expression values for a given experimental condition, which in our case was a vector of expression values in a compendium of expression profiles for a certain experimental conditions. Secondly, the list of known regulation relationship between known TF, the list of genes regulated and not regulated by these TF. These lists can be constructed from publically available databases of experimentally characterized regulation like RegulationDB [10]. Although, negative examples of these TF are not well documented in databases.

Once the lists have been prepared, CompareSVM splits the problem in many sub-problems; each sub-problem was associated with TF in a list of known TF. For each TF, we trained a binary classifier to differentiate genes known to be regulated and the list of genes not known to be regulated by the TF, based on the genes expression profiles. The concept was to measure the expression level of TF and its target genes using same assumption used by many unsupervised algorithms for GRN. If two genes were regulated by the same TF then they were likely to had exhibit same expression patterns. Once the classifier had been trained for the TF on training data, the list of new genes can be assigned to given TF if their score meets the threshold. Local models were generated for each TF and new genes were assigned to them using threshold. The final step was to combine the score of all local models to rank the candidate TF-gene interaction list. A similar approach was used by Bleakley [2] and SIRENE [3]. This process was repeated for four different types of kernels to give comparative results.

### Evaluation

We performed evaluation on simulated, steady-state expression data, generated from sub network from *E. coli* network, in order to check the accuracy of an algorithm against true known network [11]. We used GeneNetWeaver [12] to extract and simulate the gene expression data. GeneNetWeaver provides methods for both *in silico* benchmark generation and performance profiling of network inference algorithms. GeneNetWeaver extracts sub networks from the list of known interaction networks such as *E.coli* and *S.cerevisiae*. It emulates transcription and translation using set of ordinary differential equations to generate expression data for knockout, knockdown, dual knockout and multifactor experiments.

The expression value of a given gene is set to zero in simulation of knockout experiment, whereas in knockdown experiment, the expression value is halved. In case of multi-factorial experiments, the expression values of small number of genes are perturbed by a small random number. We only provided expression data and did not provide metadata information to algorithm like in DREAM challenge. Unsupervised methods are only provided with expression data, whereas for supervised methods, interaction data is provided in addition to expression profiles. Unsupervised methods were used with their default parameters and for supervised methods five cross validation was applied and parameters were optimized on training data only.

## Results

We evaluated the prediction accuracy of unsupervised method (CLR) using estimation (Pearson, Spearman) with comparison to supervised approaches (SVM using four different kernels that have been mentioned in the methods section) using microarray simulated data. The prediction accuracy was measured by the area under the Receiver Operator Characteristics curve (AUC) for prediction methods (supervised and unsupervised) using three different experimental conditions (knockout, knockdown and multifactorial) and also average of three experimental conditions (all). Networks of node size 10, 30, 50, 100, 150, 200 and 500 were extracted from *E. coli* and expressed data were simulated by GeneNetWeaver. GeneNetWeaver generated number of nodes equal to number of experiments. 10 networks were simulated and generated by GeneNetWeaver for a given size of network and experimental condition, therefore each evaluation was tested ten times. The large standard deviation was observed in predication accuracy of all methods and experimental conditions. For small network, the accuracy varies from random networks to close perfect prediction.

In unsupervised method CLR, the correlation method (Spearman, Kendall) performed poorly for knockdown experimental condition. In knockout experimental condition, same pattern was observed, but as the network size approached to 500, the predication accuracy approached to 70% as shown in Figure 3. In multifactor experimental condition, it performed exceptionally well and slightly better than supervised methods. But its prediction performance was not suited for network with small number of nodes even in multifactorial experimental condition as shown in Figure 3.

CLR, the correlation method Pearson has more dramatic impact on prediction accuracy on knockout experimental condition compared to Spearman as changes in the expression values are not monotonically distributed, but both performed poorly on the knockdown experimental condition with comparison to SVM. Pearson performed very well as the size of network increased as well as on the network with few nodes compared to Spearman correlation method. Although, supervised methods had higher prediction accuracy for small network, but as the size of network increased in multi-factorial experimental condition, the CLR (Pearson, spearman) performed better as shown in Figure 3.

In SVM, the three (linear, Gaussian and poly) out of four kernel methods outperformed the unsupervised methods with the exception of multifactorial experimental condition. Although, for network size up to 200 nodes, performance was better than unsupervised methods in multifactorial condition as well. In all experimental conditions, the Gaussian kernel was most consistent and had least standard derivation. Polynomial kernel achieved highest accuracy in knockout experiments as network size approached to 500 nodes and in knockdown experimental condition, linear kernel marginally better than polynomial kernel in prediction accuracy for large networks as shown in Figure 3. In multifactorial condition, these three kernels (linear, Gaussian and poly) performed well for networks of small size up to 200 nodes and slightly underperformed as network size approached 500 with compared to unsupervised methods. Sigmoid kernel had poor performance in all experimental conditions and its performance was not better than random guess.

**Figure 4:**
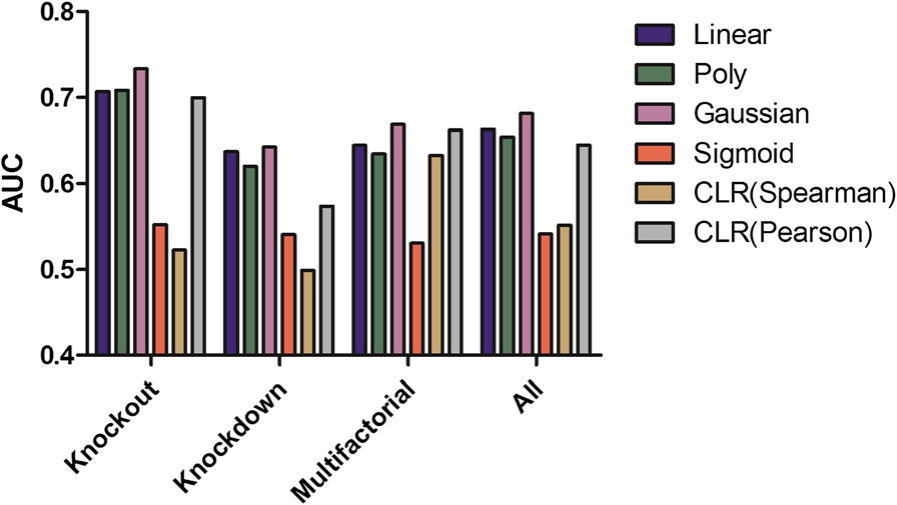
**Prediction accuracy (AUC) average overall network sizes of unsupervised and supervised methods on knockout, knockdown, multifactorial and average data generated by GeneNetWeaver and extracted from** ***E. coli.*** For each network of size: 10, 30, 50, 100, 150, 200 and 500. 10 networks were generated for each size and experimental condition.

Gaussian kernel possibly is the best option for prediction of GRN from microarray data as it has high accuracy and less standard derivation on small datasets compared to all other inference methods. It also has overall best performance for all biological conditions (Figure 4).

Linear kernel is a fast and does not require any additional parameter to optimize the prediction with contract to all other kernel methods. Even with its simplicity compare to other complex kernels, it still performs almost same as other complex kernel methods and ranked 2^nd^ in overall prediction accuracy. It slightly outperformed the Gaussian kernel as the size of node reaches 500 in three biological experimental conditions.

Our analysis indicates that suitability of a method for predication of GRN depends on biological condition and size of network. For small networks (<200), all biological conditions can be inferred by Gaussian kernel with high prediction accuracy (Figure 4). As the size of network approaches to 500, the CLR (Pearson, Spearman) outperforms the all other methods in multifactorial condition, but SVM (poly nominal kernel) is best suited for knockout data and SVM (linear kernel) for knockdown. Gaussian overall performed better in all experimental conditions and sizes of the network as shown in Figure 4.

## Discussion

We have developed a tool based on SVM to infer GRN, and used it to compare four widely used kernels to evaluate prediction accuracy on three biological conditions and combination of all. Our results are in agreement with already published report [2], but as the size of nodes exceeds 200, the unsupervised method (CLR) slightly outperforms the supervised method (SVM) in multifactorial experimental condition. We have also observed that the large number of repeats is required on network of different sizes to accurately estimate the prediction accuracy of these methods.

The most important observation from this evaluation is that there is no one universal method suitable for inference of GRN for all biological conditions. The suitability of inference method depends up size and type of expression profiles of microarray data. On average, the unsupervised methods achieve low accuracy with exception of multifactorial dataset. Although, Pearson correlation is comparatively accurate enough and even does not require parameter optimization. Unsupervised methods are suitable for simple and small problem but are fast and do not require any prior training for inference of GRN.

The limitation of CompareSVM due to its supervised nature approach is inability to predict target of TF which have no prior known targets. The performance of the CompareSVM depends upon the list of known target genes; therefore, TF with incomplete list of interaction will produce poor local models. This may direct the research into hybrid model which are based on supervised and semi-supervised methods which can address this challenge.

## Conclusion

To summarize, CompareSVM can be used to infer GRN with high accuracy (AUC) for networks (<200) with SVM Gaussian kernel for biological datasets (knockout, knockdown, multifactorial and all). For large network, choice of algorithm depends upon the type of biological condition. There had been variation in prediction accuracy in all inference methods, therefore; prediction should be limited for simple network. Future work is needed for the development of semi-supervised methods that have the ability to predict target of TF which have no prior known targets.

## Additional files

Additional file 1:
**Software package. CompareSVM.zip file that contains MATLAB scripts and example datasets to demonstrate our approach.**
Additional file 2:
**ReadMe: A world file containing detailed manual to install and run the software.**
Additional file 3:
**A zip file contains dataset (expression profiles) ranging from 10 to 500, each experiment type and size has 10 different expression profiles.**
Additional file 4:
**A zip file containing results of sample runs for three examples.**
Additional file 5:
**A zip file contains R script for unsupervised algorithm CLR.**
