# Supplementary material for: CompareSVM: supervised, Support Vector Machine (SVM) inference of gene regularity networks
Source: BMC Bioinformatics. 2014 Nov 30;15(1):395. doi: 10.1186/s12859-014-0395-x (PMC4260380; doi:10.1186/s12859-014-0395-x)
Supplement: Additional file 2: — ReadMe: A world file containing detailed manual to install and run the software. [file 12859_2014_395_MOESM2_ESM.docx]

**Manual for MATLAB interface of CompareSVM**

CompareSVM provides a simple interface to predict gene regulatory network (GRN) from microarray data sets. Detailed data for testing can be downloaded from our website based on simulated data generated by GeneNetWeaver.

**Contents**

1. Installation
2. Preparation of dataset
3. Work flow
4. Sample Results
5. Unsupervised methods in R

**1 Installation**

- 1. install MATLAB
  2. Download LIBSVM (<http://www.csie.ntu.edu.tw/~cjlin/libsvm/>)
  3. Install LibSVM version 3.1.7 (Chih-Chung Chang and Chih-Jen Lin)
     1. unzip the package
     2. Add path of folder of LibSVM to MATLAB
     3. pre-built binary files are already for 32 bit (skip 1.3 section if you are using 32 bit windows)
     4. type make to generate and build svmpredict.mex, libsvmread.mex and svmtrain.mex (if above installation fails please type mex –setup )

***for more details please read libsvm readme file ***

***if libsvm is not properly as it uses external compiler CompareSVM will not be able to run and files will give errors

- 1.
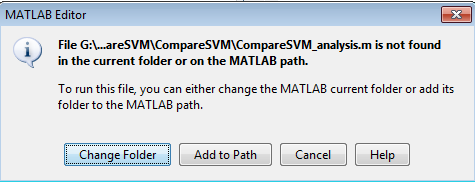
CompareSVM folder and its subfolders are required to be added to MATLAB path.

1. **Preparation of dataset**
   1. Simulated dataset can be generated using GeneNetWeaver (network sizes ranging from 10 to 500 of all three experimental conditions are attached in supplementary data). These file are required to be named according to be read by CompareSVM. GeneNetWeaver can be download from (<http://gnw.sourceforge.net/genenetweaver.html>)
      1. expression file is to be named as expression.tsv in folder CompareSVM/dataset/expression .tsv
      2. regulation file is to be named as regulation.tsv in the folder CompareSVM/dataset/regualtion.tsv
   2. Actual dataset can be generated by using microarray data and regulation files using different interaction databases.
   3. Format of expression and regulation files
      1. **Expression file**: first row should contain list of all gene and rest of rows should represent result of each individual microarray experiment profile as shown in figure below.


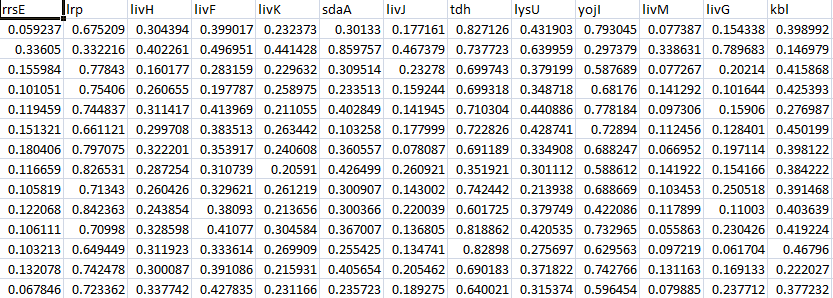


- - 1.
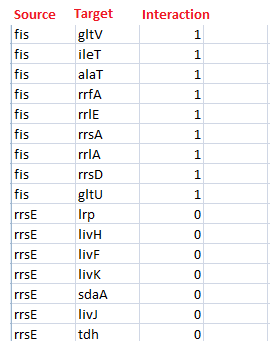
**Regulation file** : It should have 3 columns, 1^st^ column should represent source gene, 2^nd^ column should represent target genes and last column should contain interaction details( 0 if there is no interaction and 1 if there is interaction) as shown in figure below

1. **Work flow**

The tool provides simple interface to use SVM for inference gene regularity network, CompareSVM is divided into three parts as follow

- parameter optimization (CompareSVM_optimzation)
- comparative analysis (CompareSVM_analysis.m)
- predication (CompareSVM_prediction)
  1.
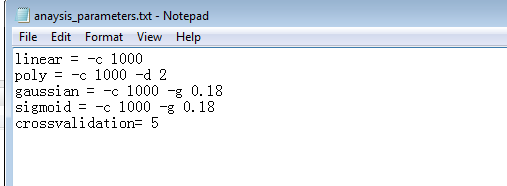
analysis_parameter.txt (CompareSVM/dataset/analysis_parameter.txt) contain set of parameters for 4 kernel methods, it require C parameter for all kernel methods, additional parameter for polynomial, Gaussian and sigmoid kernel according. 1 addition parameter for cross validation is also required. For new species, these parameter can be optimized using optimization (details in optimization section of CompareSVM).

**Options**:

-c cost: set the parameter C

Cross validation minimum require 2


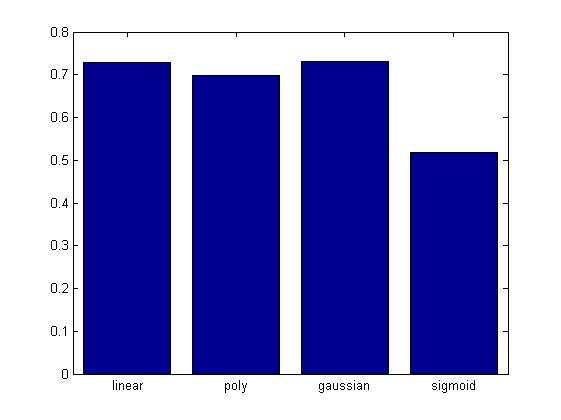
**Sample output**

- 1. optimzation_parameters.txt (CompareSVM/dataset/ optimzation_parameters.txt) requires only two parameters, choice of kernel and cross validation. It uses grid search to return optimized parameter. We used *E coli* dataset to find optimized parameters using large scale simulations on different size of networks.

***If optimized parameters are known than skip this section. A grid search is used to fine the optimized parameters, prior knowledge can be used to save time ******


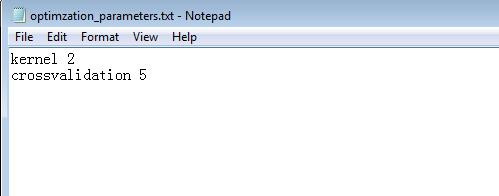


**Options**:

Kernel (range from 0 to 3)

0 -- linear: u'*v

1 -- polynomial: (gamma*u'*v + coef0)^degree

2 -- radial basis function: exp(-gamma*|u-v|^2)

3 -- sigmoid: tanh(gamma*u'*v + coef0)

Crossvalidation minimum require 2


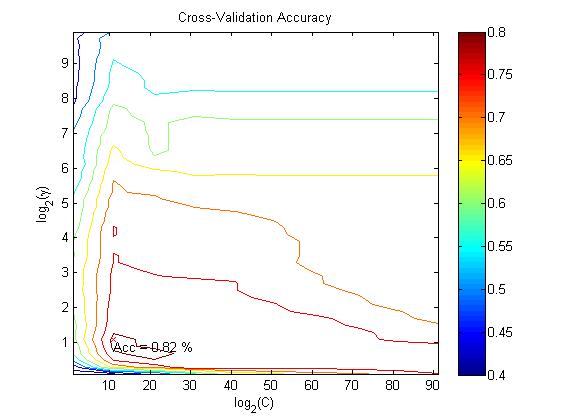
 **Sample output**

- 1. Once Optimized parameters have been known using CompareSVM_optimzation and appropriate kernel has been selected with CompareSVM_analysis.CompareSVM_prediction canbe used by providing the parameter to prediction_parameters. It will return a matrix in xls format, where columns will represent TF and column will represent list of genes. sample parameters


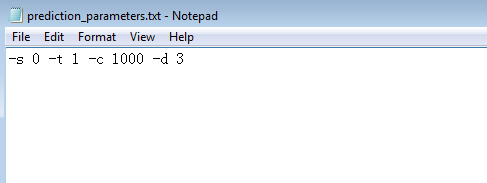


**Options**:

-s svm_type : set type of SVM (default 0)

0 -- C-SVC

1 -- nu-SVC

2 -- one-class SVM

3 -- epsilon-SVR

4 -- nu-SVR

-t kernel_type : set type of kernel function (default 2)

0 -- linear: u'*v

1 -- Polynomial: (gamma*u'*v + coef0)^degree

2 -- Radial basis function: exp(-gamma*|u-v|^2)

3 -- Sigmoid: tanh(gamma*u'*v + coef0)

**** -d will be used in case of polynomial kernel, -g will be used in case of gussian and sigmoid kernel. ****


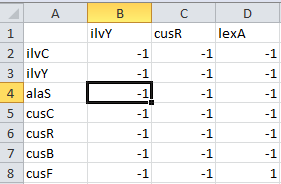
**Sample output**

CompareSVM_prediction generates xls file, where columns and rows represent each gene name , each cell of file contains either 1 or -1, 1 represent there is interaction between genes and otherwise

- 1.
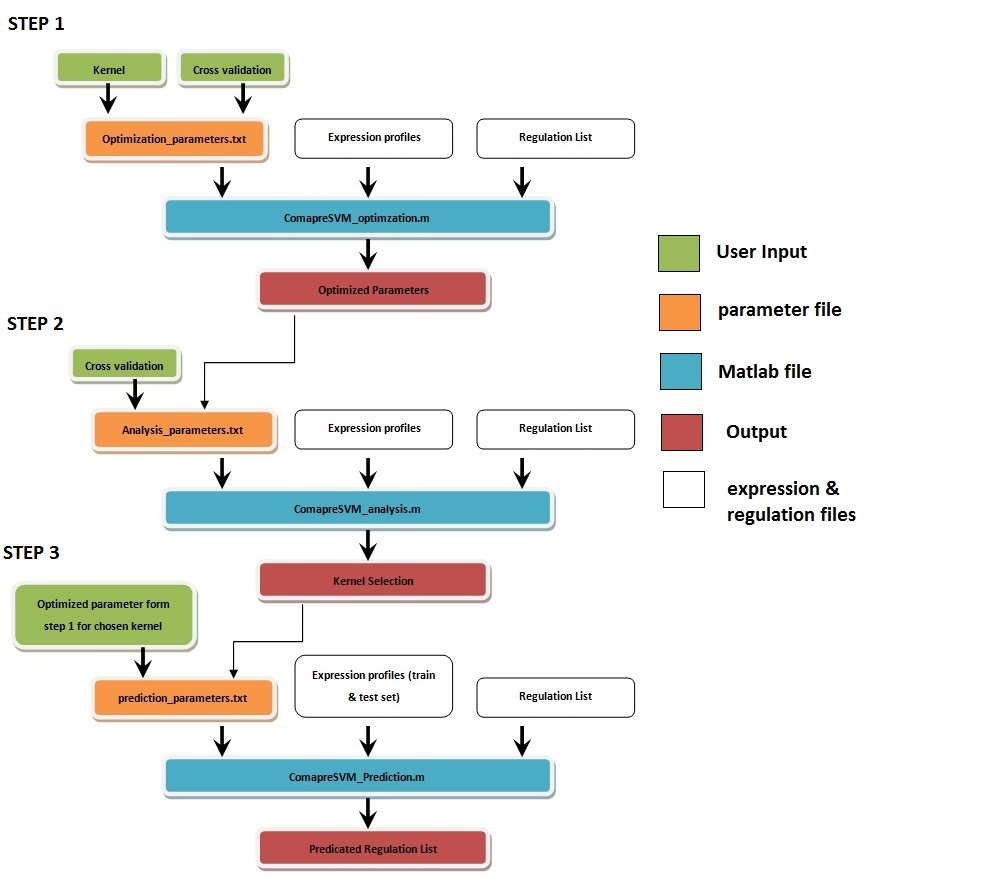
Typical work flow is shown below

1. **Result examples**

A supplementary file2 has been added to supplementary data, this file contain 3 runs on E-coli on the network of 150 genes, each data set was run using different kernel function. All the input files and output generated files have been shown in the same supplementary file2. A complete list of datasets are also available in supplementary file1.

**5 Unsupervised methods in R**

Unsupervised method CLR, the correlation method (Spearman, Kendall) was run using R package. The script file in R is included in supplementary file3.
